# Polyurethane Recycling and Disposal: Methods and Prospects

**DOI:** 10.3390/polym12081752

**Published:** 2020-08-05

**Authors:** Aleksandra Kemona, Małgorzata Piotrowska

**Affiliations:** Institute of Fermentation Technology and Microbiology, Faculty of Biotechnology and Food Sciences, Lodz University of Technology, Wólczańska 71/173, 90-924 Łódź, Poland; malgorzata.piotrowska@p.lodz.pl

**Keywords:** polyurethane, recycling, energy recovery, chemical degradation, microbial degradation, enzymatic degradation, polyurethane modification

## Abstract

Growing water and land pollution, the possibility of exhaustion of raw materials and resistance of plastics to physical and chemical factors results in increasing importance of synthetic polymers waste recycling, recovery and environmentally friendly ways of disposal. Polyurethanes (PU) are a family of versatile synthetic polymers with highly diverse applications. They are class of polymers derived from the condensation of polyisocyanates and polyalcohols. This paper reports the latest developments in the field of polyurethane disposal, recycling and recovery. Various methods tested and applied in recent years have proven that the processing of PU waste can be economically and ecologically beneficial. At the moment mechanical recycling and glycolysis are the most important ones. Polyurethanes’ biological degradation is highly promising for both post-consumer and postproduction waste. It can also be applied in bioremediation of water and soil contaminated with polyurethanes. Another possibility for biological methods is the synthesis of PU materials sensitive to biological degradation. In conclusion, a high diversity of polyurethane waste types and derivation results in demand for a wide range of methods of processing. Furthermore, already existing ones appear to be enough to state that the elimination of not reprocessed polyurethane waste in the future is possible.

## 1. Introduction

**Figure 1:**
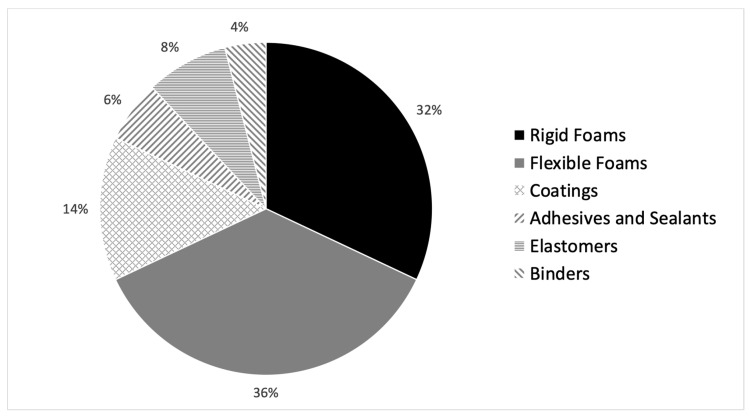
Market share of different types of polyurethane products.

Production of synthetic plastics started in the first decades of the nineteenth century. The relatively low production costs resulted in their presence in almost every area of life, which led to the current annual production of up to 360 million tons. Polyurethanes (PU) represent almost 8% of produced plastics which place them as the 6th most used polymer in the world [1]. PU’s are generally classified into two groups: foams and denominated CASE’s (Coatings, Adhesives, Sealants, Elastomers) [2]. Furthermore, there are two types of foams: flexible (that are applied in mattresses and automotive seats) and rigid (applied in buildings isolation and commercial refrigerators). CASEs are mostly used as parts of sports shoes, athletics tracks, electronic products and ships structures. The final polyurethane products market in EMEA (Europe, Middle East and Africa) is almost equally divided between flexible foams (36%), rigid foams (32%) and CASE (32%), as is illustrated in Figure 1 [3].

In 2017, total production of polyurethane products in EMEA was reported as 6.47 million tons. Most of this production is placed in Western Europe (more than 3.6 million tons in 2017). However, the fastest growth was presented by Eastern Europe, mostly due to strong growth in Poland and Turkey.

The versatility of polyurethane foams and their susceptibility to modification allows them to replace several of previously used materials, of synthetic (PVC, rubber, polystyrene), and natural origin (leather) [4,5,6,7]. Such action can be dictated by the cost of production of traditional plastics such as polystyrene, which are higher than in the case of polyurethanes. Some of them can have significantly lower risk of release of toxic volatile organic compounds like waterborne polyurethane coatings in comparison to traditional one using solvents [8]. Sometimes obtained materials are characterized by significantly improved mechanical properties (greater durability than PVC), high resistance to solvents such as water (they are only raw materials that can be used in submarines coatings), oils and organic solvents, improved adhesive or electrical properties, etc. [9,10]. Table 1 lists the main advantages of PU against rubber, metal and plastic.

However, grooving production of polyurethanes, which are mostly applied in products that wear out over time (like clothes or shoes), or that are exchanged for different or better ones (like cars, furniture or sports equipment), leads to accumulation of post-consumer garbage. Due to PUs low susceptibility to physical, chemical and biological factors, and toxicity of some of the combustion products, landfilling is still the most common way to process polyurethane waste.

However, in recent years, polyurethane waste processing gained importance all over the world, because of the depletion of world reserves of fossil fuels such as petroleum, and the decreasing availability of landfill space.

Additionally, not without significance are legislative changes such as modification of Directive 1999/31/EC about landfilling by the European Commission. According to the revised regulations after 1 January 2015, only 25% of municipal waste in comparison to the previous year can be deposited in the landfill, and since 1 January 2030 this amount will not exceed 5%. Besides, the percentage of recycling and incineration should be significantly improved [1,11].

This paper is an overview of the literature relating to polyurethane waste processing, in particular: landfilling, mechanical processing, chemical recycling, energy recovery and biological methods. It also includes an analysis of possible applications of those methods.

## 2. Polyurethanes

**Figure 2:**
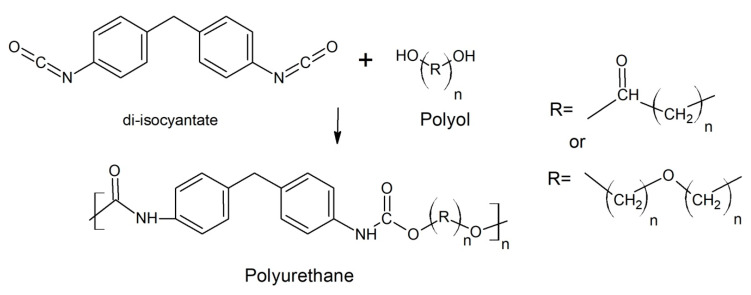
Polyurethane synthesis reaction example.

Polyurethanes are one of the most versatile polymers available for industry. Opposite to most popular polymers like polypropylene or polyethylene, polyurethanes include no polymerization products but are classified as condensation polymers [12]. Raw materials most commonly used for the synthesis of PU are polyisocyanates (usually methylene diphenyl diisocyanate (MDI) and toluene diisocyanate (TDI)), and polyols (Figure 2). More than 70% of the currently used polyols are polyetherols, polyalcohols produced by the polymerization of propylene and ethylene oxides [13,14]. Properties of the polyols and thus the properties of polyurethanes vary depending on the number of hydroxyl groups, the molar mass and the percentage of groups derived from ethylene oxide. A wide variety of substrates generate lots of material types of very diversified properties [15].

As condensation polymers, PU’s are not built of repeatable identical monomers but consists of different segments connected by various chemical bonds. Their most important unit is urethane bond (–NH–COO–), which is formed by the reaction of an isocyanate group (–N=C=O) with an alcohol group of the polyols.

Besides the two main raw materials, a variety of additives such as foaming agents, fillers and flame-retardants are used. Foaming agents are methylene chloride or carbon dioxide, which are formed by the reaction of isocyanates with water [16].

The water-blown PU foams contain a significant number of urea bonds, which are formed by the reaction of water molecules with an isocyanate group, following the reaction of newly created amine with another isocyanate group. An important feature of polyurethanes is the presence of alternating rigid (isocyanate) and flexible (polyol) segments. This allows their usage as the elastic elements of furniture and mattresses, lasting coatings, lightweight and durable construction materials and elegant artificial leather.

## 3. Polyurethane Waste Management

Due to their varied applications and commercial success, an increasing quantity of polyurethane waste is produced every year. Such waste comprises end-of-life (EOL) and post-consumer (PC) products as well as scraps from polyurethanes manufacturing. The latter is the result of production and processing methods’ imperfections and can make up to 10% of produced PU’s [17]. However, EOL and PC waste is a much bigger problem, because they are usually contaminated or deformed, and are therefore less prone to being reused [18].

**Figure 3:**
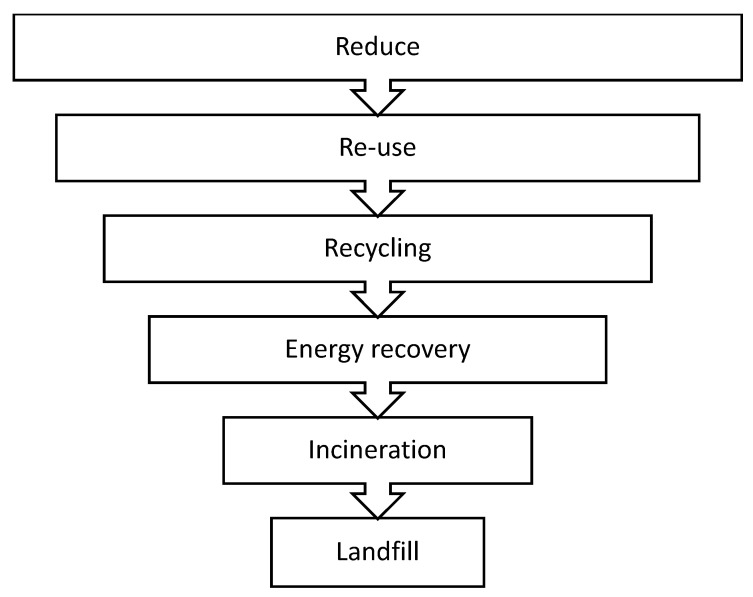
Ladder of Lansink.

Solid waste management is usually governed by the “Ladder of Lansink” (Figure 3), which determines a generally accepted hierarchy of methods for dealing with waste and was the basis for the hierarchy in force from Directive 2008/98/EC of the European Parliament and of The Council of 19 November 2008. This hierarchy lays down a priority order in waste legislation and policy. However it admits the possibility of omitting some of steps if it is necessary due to technical economical or environmental reasons [19].

### 3.1. Landfilling

Landfilling is still the most common way to process polyurethane waste. The fraction of PU’s disposed this way reaches almost 50% of waste, (combined postconsumer or postproduction ones) [20]. As the polyurethane foams have the greatest share of production, they also are the biggest problem. Due to their low apparent density, they have large volumes. Furthermore, a great amount of air contained inside foam cells can provide oxygen for deep-seated fires and impede efforts to extinguish flames. Another hazard related to landfill fires is toxic fumes, produced during polyurethane combustion. Recycling is a great alternative to landfills, but despite a great effort from producers and legislative units, it is still not a predominant method of PU waste disposal conduct [21].

Concepts of Enhanced Waste Management and Enhanced Landfill Mining aim to reclassify landfilling as a balanced proceeding. Landfill should be considered a temporary storage place for wastes waiting for valorization and further processing, rather than a final solution. Depending on their state and available technologies, stored materials can be used as a stock for new products (Waste-to-Product) or as energy source (Waste-to-Energy) [22].

### 3.2. Mechanical Recycling

Mechanical processing is the easiest and most basic way to recycle PU’s. It involves a change of solid waste into flakes, granules or powder. They can be used directly as filling for pillows, toys, etc. (Primary Mechanical Recycling) or as a substrate in subsequent processes (Secondary Mechanical Recycling and Feedstock recycling).

Fragmentation can be achieved by grinding, cutting or tearing. Preparation of fine powders (with particles less than 100–125 microns) employs two-roll mills processing. Obtained powders can be used as fillers in newly manufactured polyurethanes. The viscosity of the PU foam is the main limitation of this technique. Furthermore, this technique is relatively uneconomical, and acquired products are of limited quality, which greatly limits the available sales markets. Preparation of powders with a larger diameter (less than 250 microns) uses precision knife cutting. Granules are prepared with pellet mills. They consist of two or more metal rollers, which press the polyurethane through the metal plate with holes. Shredded foams can be used as a feedstock for two types of reprocessing depending on the usage of adhesives. Post-consumer waste products cannot be the stock for mechanical recycling due to their contamination or the addition of other materials [23,24].

#### 3.2.1. Mechanical Reprocessing with Adhesives

Re-bonding involves connecting a great amount of polyurethane foam scraps or pellets with the adhesive, which usually consists of a polyurethane compound. Used particles have a diameter of a few millimeters. The density of products obtained in this process is higher than of source materials. They have properties that are hard to achieve with standard PUF production methods. Resulting materials, due to its durability and flexibility are used as floor coverings and sports matting. Annually about 90% of flooring underlay market comes from re-bonding processes [25].

Adhesive pressing processes pellets with the addition of adhesive (usually polymeric isocyanate), with high temperature and pressure (30–200 bar and 100–200 °C). This process allows the preparation of contoured products such as car mats or covers for tires. Polyurethane building panels produced by this method show high moisture resistance and excellent insulating properties. The main limitation of this method is the usage of flame-retardants in many types of foam, which considerably reduces the available raw materials stock and requires the segregation of waste. Furthermore, their high density limits potential markets [23].

#### 3.2.2. Mechanical Reprocessing without Adhesives

Hot compression molding is one of the processes that do not require binders. Finely ground particles of polyurethane foam are condensed under high pressure and temperature (180 °C and 350 bar). This technology is mainly used in the processing of rigid polyurethane foam waste from the automotive industry such as bumpers. Gained products have a rigid structure and are three-dimensional (ready-for-application parts such as pump and motor covers), and when strengthened by fiberglass during molding, they can be used as dashboards, door panels etc. Materials that are created with polyurethanes as fillers during compression molding of different resins (such as polyester resin mix), display increased flexibility and impact toughness over the mineral-filled materials. Two main disadvantages of these materials are the problematic recycling of painted parts and difficulties with producing smaller particles needed in molded products [26,27].

Reaction injection molding (RIM) is a technique applied in the production of electrical covers, computer end telecommunication equipment enclosures. This process involves mixing two liquid components (polyol and isocyanate) and injecting them into a preformed mold. The chemical reaction results in the formation of polyurethane in the desired shape. It can be uniform or foamed and may be reinforced by fiberglass (RRIM) or a structural composite (SRIM). RIM can be applied for mixed polyurethane batches and its mixtures with other plastics (usually thermoplasts). Rigid PU foam waste, after regrinding into a fine powder, can also be used as a core [23,28].

### 3.3. Chemical and Feedstock Recycling

Feedstock recycling, also called tertiary, is a type of polymer reprocessing, which leads to the conversion of polymer chains to smaller molecules through chemical processes. Amongst them are hydrolysis, glycolysis, aminolysis, gasification and others. An important feature of the polyurethanes is the possibility of reversing the polymerization that allows for the recovery of building blocks. Considering costs, applied temperature and additional substrates, chemical recycling is much more demanding than mechanical. It allows breaking down polymers into various raw materials that can be utilized either as resources for processes unrelated to polyurethane production and processing or as a stock material for new PU synthesis.

#### 3.3.1. Hydrolysis

**Figure 4:**
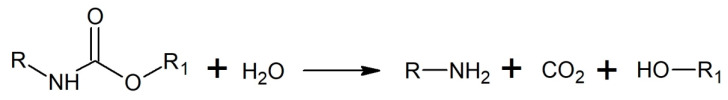
Polyurethane hydrolysis.

Hydrolysis was the first chemical method developed to recycle polyurethane waste, especially flexible foams. It is a reaction between polyurethane waste and water, that can be liquid or in the form of steam (Figure 4). The resulting compounds include polyols, amine intermediates and carbon dioxide [29].

Its most important advantage is the possibility of application for both production scraps and postconsumer waste [23]. The process is conducted in an anaerobic environment and at high temperature (above 150–320 °C). The resulting polyols can be used as additives to the original polyol in polyurethane production or as fuel. Furthermore, amine intermediates after treatment with phosgene enable the recovery of the starting isocyanates. Reacting polyols and isocyanates obtained from hydrolysis allows producing polyurethanes that made up inset [30]. Biggest disadvantage of hydrolysis is that it requires high energy input into reactor either to heat up the batch or to apply high pressure, which renders this process as uneconomical. Due to this reason hydrolysis still has not been translated into a commercial scale [12].

#### 3.3.2. Hydroglycolysis

Hydroglycolysis is an improvement of hydrolysis, in which water and glycols are combined under less demanding operating conditions. The reaction proceeds in the presence of lithium hydroxide as a catalyst at a temperature of 200 °C. Its great advantage is the high tolerance to contamination and heterogeneity of the used polyurethane feedstock; it is not commonly used due to the high cost of the process. The resulting polyols may constitute up to 50% of a mixture with virgin starting material for the synthesis of polyurethane foams [12,23,31].

#### 3.3.3. Aminolysis/Ammonolysis

**Figure 5:**
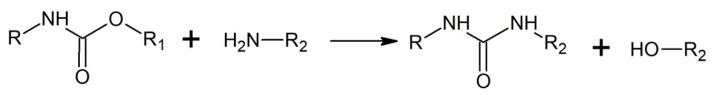
Polyurethane aminolysis.

Aminolysis is a transesterification reaction, in which the amine group from ammonia or amine interchanges the ester group from urethane (Figure 5). Materials containing polyurethanes are mechanically ground or dissolved in a suitable solvent like cyclic ether, or chlorinated hydrocarbon solvent containing nitrogen. Later they are subjected to aminolysis with a compound containing at least one –NH_2_ group.

The temperature of this reaction is between 80 and 190 °C, and it usually is conducted under inert gas with catalysts such as sodium hydroxide, aluminum hydroxide and sodium methoxide [32]. Depending on used diamine or amino alcohol it is possible to obtain bi- or polyfunctional amines and alcohols. They can be applied in the synthesis of polyurethanes, melamine resins, epoxy resins, polyesters, polycarbonates, etc. It is also possible to use two or more resulting functional phenols for the recovery of the corresponding isocyanates [33].

Aminolysis has been described only for the polyurethane foams recycling and was not developed for the denominated CASEs. It has also never been applied further than the research stage [12].

#### 3.3.4. Phosphorolysis

**Figure 6:**
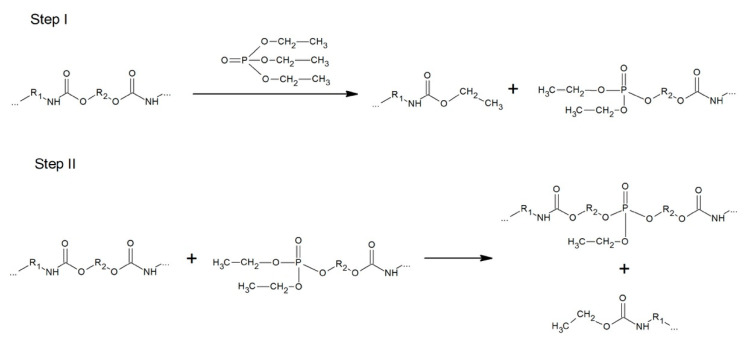
Polyurethane phosphorolysis.

Processing polyurethanes with esters of phosphoric and phosphonic acids leads to the conversion of the wastes into a liquid product, which is a result of the reaction between the urethane group and the ester alkoxy group. This process allowed obtaining a mixture of phosphorus-containing oligouretanes (Figure 6). It can be used in the production of new polyurethanes with improved flame retardancy (similar to commercial products containing higher amounts of flame retardants), adhesive properties and UV resistance. The disadvantage of the process is that it is not possible to recover the input materials so that they cannot be recycled back to the process but are merely moved to other types of production [34]. Moreover, there has been no technical scale application of phosphorolysis, and there have been no reports about progress in this topic since 2013 [12].

#### 3.3.5. Glycolysis

At present, glycolysis is the most used chemical recycling method for rigid and flexible polyurethane. It is based on a transesterification reaction in which a hydroxyl group from glycol replaces the ester group containing a carbonyl carbon of a urethane bond. This reaction produces polyols for which the properties can be controlled to some extent, and may be similar to those of original material. They can be used in the production of polyurethanes [35,36].

There are two main directions of the glycolysis. The first one leads to the recovery of polyols for the production of flexible polyurethane foam. Second, so-called split-phase glycolysis (SPG) results in rigid and flexible polyols. SPG is based on separate processing of top and bottom layers from diethylene glycol (DEG) glycolysis. The upper layer, mainly comprising the flexible polyols, is washed with DEG, and the lower is treated with propylene oxide resulting in the formation of rigid polyols.

The main limitation of glycolysis is the difference in process parameters for flexible and rigid foams, which enforce segregation of used waste. This method is also significantly more effective when applied to the post-production waste due to the high sensitivity of the reaction to the presence of impurities that may generally occur in consumer waste [23,37].

#### 3.3.6. Gasification

Gasification is a highly exothermic reaction of partial oxidation of carbonate materials. Its main products are “syngas” (a mixture of mainly carbon monoxide and hydrogen) and ash. This is a highly exothermic reaction. One of the most significant advantages of gasification is the absence of the need to segregate waste. Besides, polyurethanes mixed with other raw materials may also be used in the process. However, the economy of this process is considerably dependent on the potential use of syngas as a source of energy and raw material for the synthesis of methanol, ammonia, carbohydrates, acetic acid, etc. Moreover, the use of atmospheric air as a reaction medium simplifies the process but molecular nitrogen tends to reduce the energy obtained during gasification. According to research, gasification processes produce significant quantities of toxic hydrogen cyanide and nitrogen dioxide. The addition of a suitable catalyst can reduce this emission to some extent. The presence of oxygen also contributes to reducing the amount of waste generated as a result of secondary reactions to nitrogen compounds present in ashes [38].

#### 3.3.7. Pyrolysis

Pyrolysis is the process of thermal decomposition of long polymeric chains into less complex molecules under anaerobic conditions at elevated pressure. Main products—oil, gas and ash—can be valuable for many industries [39]. As demonstrated by Jomaa et al. in 2015, the polyurethane pyrolysis progresses in at least two steps, which correspond to the subsequent decomposition processes of the polyol part and the isocyanate part. The first stage operates at a temperature range of 100–300 °C and over 50% of the polymer mass is lost. Between 300 and 800 °C begins the second stage of decomposition. Ash remaining after pyrolysis is less than 3% of starting polyurethane [40]. Garrido and Font investigated the pyrolysis of flexible polyether polyurethane foams [16]. Their results confirm the two-step degradation of these polymers, indicating that the urethane bond breaks down in the first step, resulting in isocyanates, while in the second step the decomposition of the ethereal polyol results in the formation of ashes similar to those formed in the combustion process. An undoubted advantage of pyrolysis is the small amount of waste remaining after the process and the possibility of using the resulting products in other petrochemical processes. Unfortunately, this is not always possible due to the inability to quantify the ratio of individual products and the difficulty of obtaining products with the desired properties. The resulting gas is highly calorific and sometimes can be used in gas engines for heat and power. Besides, gas products contain certain amounts of toxic compounds such as hydrogen cyanide, benzene or aniline [41].

#### 3.3.8. Hydrogenation

Hydrogenation is a process similar to both pyrolysis and gasification. It leads to the formation of gas and oil. The fundamental difference between this process and pyrolysis is the utilization of high-pressure hydrogen instead of inert gas. For this method to be useful, it requires addressing two important issues as in the previous process—the composition of the obtained oil and gas and the cost of their conversion to functional products—which can then be used as energy materials and substrates in chemical processes [1].

Amongst chemical methods of polyurethane recycling, only glycolysis and gasification were implemented on a large scale, while others remained in the research stage. Moreover, only glycolysis leads to the recovery of raw material (Table 2).

### 3.4. Energy Recovery

Energy recovery is applied only for polyurethanes that cannot be processed by any recycling method because there is a greater gain in raw materials recovery. There is no possibility to restore materials from combustion and incineration processes. The most important advantage of this process is the possibility of application for polyurethanes that are contaminated, foamed with Freon or permanently linked with wood, leather or fabric. Moreover, combustion and incineration lead to volume reduction of the landfilled waste up to 99%.

This method is not flawless, especially as more and more polyurethane foams contain flame-retardants, applied to increase users’ safety. Those additives make it difficult, or even impossible, to carry out energy recovery. Furthermore, when high temperatures are applied PU foams can release toxic compounds and carcinogens, like carbon monoxide, hydrogen cyanide and nitrogen oxides [42]. Polyurethanes show much higher toxicity during thermal degradation under aerobic conditions. The amount of emitted nitrogen oxide under aerobic conditions is well above acceptable levels (up to 2.5-fold), while in the case of pyrolysis only slightly exceeds the threshold at 550 °C, and higher temperatures standards are maintained [24]. The gases generated by the combustion may also include isocyanates, which are compounds with high toxicity. Exposure to them can lead to skin, eyes and respiratory system irritation. The last one can be so strong that even 0.02 ppm exposition can lead to so-called isocyanate asthma. Higher levels can cause pneumothorax [43].

There are studies aimed at the determination of incineration conditions that would reduce or even eliminate the toxic compounds during energy recovery. Unfortunately, it is a quite tedious process. In higher temperatures there are fewer polychlorodibenzeno-*p*-dioxins and furans, but instead, the load of N-containing compounds such as NO and HCN reaches higher levels [44]. Even with those obstacles, the serious argument for the increase in the percentage of polyurethane waste administered to thermal degradation instead of landfilling is the amount of produced energy. It is comparable to coal combustion and only slightly lower than the energy obtained from fuel oil [14].

### 3.5. Biological Degradation

Biodegradation means the breakdown of organic substances by living organisms or their enzymes. It results in shortening of polymer chains and the elimination of some of its parts. That leads to the reduction of its molecular weight, and in favorable conditions, it can even result in the complete mineralization of degraded material. However, the complete degradation of larger polymers usually requires the cooperation of several different organisms. It can consist of a few stages: breakdown of the polymer to monomers, their reduction to simpler compounds and final degradation to carbon dioxide, water and methane (under anaerobic conditions).

Organic materials can undergo degradation under aerobic or anaerobic conditions. The decomposition of polymers with oxygen takes place in a natural environment, without oxygen in landfills and degradation of a mixed nature can take place in composts and soil. Landfill and compost degradation can be conducted by naturally occurring microorganisms or with the addition of a specially selected consortium of microorganisms or enzymatic mixture.

Biodegradation is usually more environmentally friendly than chemical degradation, as it does not require high temperatures and complicated reagents. Furthermore, it can be applied to the degradation of postconsumer waste [45].

There are numerous records of polyurethanes’ biological degradation in the literature [9,23,35,45]. Unfortunately, most of them are research papers that describe a limited part of this topic. They usually apply only to one kind of microorganism or polyurethane. Conversely, others cover a broad spectrum of plastics, which does not allow for a detailed analysis of every single one of them. Lots of existing papers do not distinguish the degradation of classical PU and those modified with biodegradable chain extenders or polyols. While modifications of polyurethanes applied for better degradability are very important, they do not solve problems with already existing environmental pollution. They are divided into three categories: fungal biodegradation, bacterial biodegradation and enzymatic degradation. Polyester polyurethanes are much more susceptible to biological degradation than polyether ones. Research with the application of fungal strains provides more promising results for polyether ones. This disproportion can be attributed to mechanical cracking of more recalcitrant polyether urethanes, usually applied in foams, by mycelium penetrating pores of material. On the other hand, most PU coating degradation research uses bacteria. The reason for that can be because of bacterial capacity to the formation of biofilm on the smooth coating surface (Table 3).

Decomposition of those materials can occur either as fortuitous biodegradation in the presence of other nutrients and substrates or when they are used as a source of carbon or nitrogen.

As polyurethanes consist of large particles and are known to be quite resistant, the prevailing systems for studying the degradation of polyurethanes have been utilizing microbial communities that can be found in landfill leakage water, sewage water of polymer factories and in soil.

Diverse patterns of degradation of various polyurethane samples result from specific properties of different PUs determining the accessibility of chemical bonds for enzymatic degradation, such as crystallinity, molecular orientation, or cross-linking. Amorphous regions of polyurethanes are more susceptible to degradation than crystalline regions due to their better accessibility [9]. Most of the enzymes identified as the one responsible for PU cleavage belong to the class of hydrolases.

Unfortunately, despite promising results, polyurethane biodegradation studies are still on the basic level stage. The main reason for this situation might be the long time required to obtain results [79].

#### 3.5.1. Fungal Degradation

It is known that fungi are microorganisms that are predominantly responsible for PU degradation amongst laboratory-tested species. Several works relating to the susceptibility of PUs to fungal attack can be found in recent literature. Most of the researches deal with soil microorganisms associated with degradation of polyurethanes [72,73,80]. Regardless of conditions of isolation, source of the soil and polyurethane used by scientists, most of the isolated species that show promising results in PU degradation belong to a few genres. Amongst them are *Aspergillus*, *Penicillium*, *Chaetomium*, *Cladosporium*, and *Trichoderma* (Table 3).

Almost all of the polyurethane degrading fungi are filamentous ones with definitely few positive results for yeasts [2]. Abiotic effects of fungal biodegradation can be the reason for this disproportion. Growing filaments penetrate material that leads to an increase in the pore size, which might result in material cracking. Unfortunately, the mechanism of this process and the importance of biotic and abiotic parts of fungal degradation are not fully explained. Biodegradation by fungal strains and communities was observed for all kinds of polyurethanes: polyester, polyether, thermoplastic PU, foams and coatings [69].

Khan, with his team isolated *Aspergillus tubingensis* strain, from polyurethane film buried in soil for one month. ATR-FTIR testing of polymer sample showed the disappearance of urethane carbonyl group, and the manifestation of –NH group, in comparison to the control sample [73]. A similar procedure applied by Osman and his co-researchers, led to the isolation of *Aspergillus fumigatus* strain, and ATR-FTIR results suggesting degradation in the polyester polyol region of polyurethane [72].

Six species of fungi: *Aspergillus niger*, *Penicillium chrysogenum*, and four strains of the *Cladosporium cladosporoides* complex were isolated from different environments (garden soil, decomposing PU foams collected at the municipal dumpsite, natural airflow from laboratory outdoors and fungal colonies grooving on the cold room’s PU isolation) based on their ability to degrade polyester (Impranil) and polyether (Poly-Lack) polyurethane varnish, as the sole carbon and energy source [57]. FTIR analysis of the chemical changes generated by those microorganisms showed decrement in the carbonyl signal that can be related to the attack of ester bonds from polyol fraction as well as the attack of urethane groups. Decrement observed in the CONOH bond signals serves for further confirmation of polyurethane groups being affected. Analysis of extracellular enzymatic activities of best PU-degrading fungus *Cladosporium pseudocladosporoides* after incubation on mineral medium with Impranil showed high esterase, low urease and no protease activity. That suggests that esterase is an enzyme responsible for the attack on ester and urethane groups in the PU. Moreover, Impranil degrading fungi were also able to grow on the polyether PU varnish as the sole carbon source and to degrade polyether PU foams.

Russel and his team applied a different approach in 2011, as they tested the possibility to utilize Endophytic fungi in polyurethane biodegradation. Two of the tested species, isolated from woody plants from the Ecuadorian Amazonian rainforest, both belonging to the *Pestalotiopsis* genus showed the ability to grow on polyurethane as a sole carbon source under anaerobic and aerobic conditions. Those results indicate the possibility of executing the biodegradation in compost prisms with minimal or zero interference in the natural microbiome [58].

#### 3.5.2. Bacterial Degradation

Although most of the promising results can be assigned to fungi, some researches indicate bacteria as potential polyurethane degrading microorganisms. Howard and his coworkers have been studying bacterial biodegradation of polyurethanes since the 1990s. In their studies, they identified strains of *Pseudomonas chlororaphis*, *Bacillus subtilis*, *Comamonas acidovorans*, and *Acinetobacter gerneri* as microorganisms capable of polyester polyurethane Impranil DLN degradation. They also identified enzymes responsible for this PU hydrolysis as lipases and esterases [52,53,54,79].

In 2007, Gautam and his co-researchers tested *Pseudomonas chlororaphis* in the degradation of automotive waste polyester polyurethane foam. Using the SEM photomicrographs, they imaged damage to the foam matrix by bacteria. They also checked esterase production by *P. chlororoaphis.* It suggested that in the presence of polyurethane, esterase activity could be observed. In addition, the measurement of diethylene glycol, which is one of the typical products in polyester urethane hydrolysis, the confirmed degradation of polymer [81].

Investigation of polyurethane degradation by the bacterial consortium was conducted. Microorganisms were isolated from polyurethane film buried for six months in soil collected from plastic waste disposal sites [62]. They tested bacterial consortium using the Sturm test, which resulted in almost four times more of biomass and two times more CO_2_ produced in culture with polyurethane than in control. Those results were confirmed by FTIR, indicating hydrolysis of ester bonds, and SEM images showing damage to the polyurethane matrix.

As it was with fungi, most of the research is based on soli organisms, but Nakkabi and co-researchers studied a *Bacillus subtilis* strain isolated from cedarwood. This bacterium was able to degrade Impranil in liquid Luria Bertani medium. FTIR analysis showed complete disappearance of absorbance peak related to the ester bond, which leads to the conclusion that this bond was fully hydrolyzed [50].

#### 3.5.3. Enzymatic Degradation

The main advantage of enzymatic degradation against microbial degradation is the possibility of controlled bond cleavage that leads to the generation of building blocks that can be returned to the production process or used as a substrate in the manufacturing of different materials. The main complication in this approach is the vast diversity of raw materials used in polyurethanes synthesis, which results in the need to approach every type of PU individually. Moreover, since polyurethanes do not occur in nature, the number of enzymes capable of those polymers’ degradation is highly limited. Depending on compounds used in synthesis together with isocyanate, among PU-degrading enzymes can be found oxidoreductases and hydrolases like esterases, ureases, proteases or elastase [15,82]. Most researchers using enzymes in depolymerization of polyurethanes address the degradation of thermoplastic polyurethanes or coatings (Table 4), while almost all publications about foams deal with microbial degradation.

**Figure 7:**
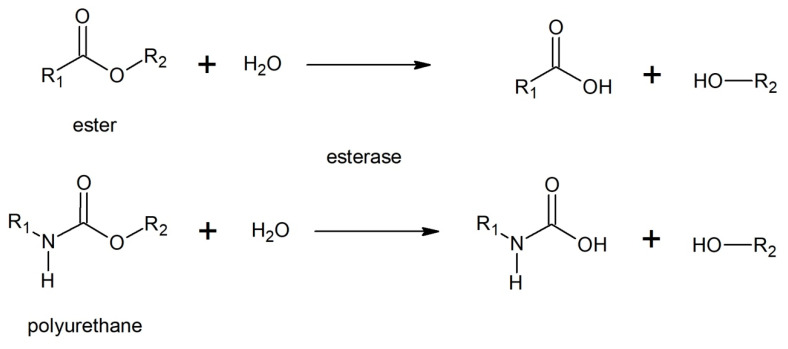
Hydrolysis of ester and polyurethane by esterase.

The most significant share of positive biodegradation results can be assigned to the hydrolysis of the polyester fraction of polyester-based polyurethanes by esterases. This reaction results in the formation of carboxylic acid and alcohol (Figure 7). Some studies show that esterases can also be responsible for the hydrolysis of urethane linkage, resulting in the presence of carbamic acid and alcohol chain-ends [103]. However, due to instability of acid which immediately breaks down, a more possible outcome is the release of amine and carbon dioxide [104]. Moreover, as most of the assays of polyurethane degradation were conducted with polyester types, especially considered as a model Impranil^®^ (Covestro, Leverkusen, Germany), they do not allow differentiation between hydrolysis of urethane and ester bonds [82]. Evaluation of urethane bond hydrolysis by esterase would be possible only if the substrate did not contain ester bonds. Studies dealing with this concept are scarce [88,97].

**Figure 8:**
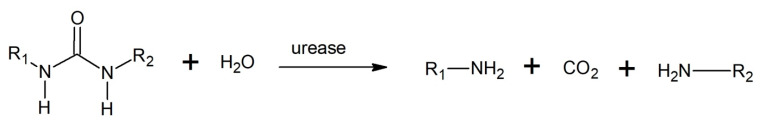
Hydrolysis of polyurethane by urease.

Researches testing PU degradation by ureases, face the same problem (Figure 8), most of them show promising results as long as polyurethane contains urea bonds [95,99,105]. Those bonds can be part of the soft segment or result from foaming with water. However, those in long, elastic chains are much easier to degrade.

**Figure 9:**
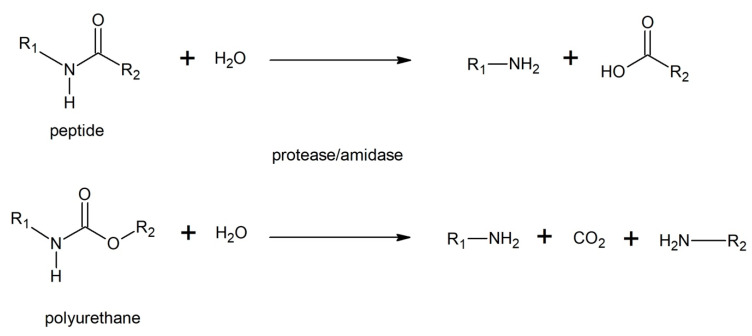
Hydrolysis of polyurethane and peptide by protease/amidase.

Amidases and peptidases also appeared to be quite efficient for urethane bond hydrolysis (Figure 9), resulting in the formation of an amine and alcohol as well as the release of carbon dioxide [15,90,94].

While Howard and his coworkers used bacteria as their starting point, they moved to single enzymes, isolated and chosen as the one with the most promising results in Impranil degradation. Enzymes isolated from *Pseudomonas chlororaphis* displayed esterase and protease activity [53,54], and those from *Bacillus subtilis* showed esterase and lipase activity [49]. A similar approach to *Comamonas acidovorans* allowed the isolation of protein displaying esterase activities [52,63].

Based on Ruiz and Howard’s research, Petry do Canto et al. [85] developed three-dimensional structures of polyurethane degrading enzymes. They used the technique of homology modeling. Their theoretical molecules showed good stereochemical quality and stability of structures in simulations. During molecular docking, all monomers of polyurethane that they tested showed favorable interactions with models.

A significant share of the polyurethane degradation research approaches this topic from a different angle, because polyurethanes have lots of medical applications like in wound dressings, tissue engineering scaffolds, implants and drug delivery with nanoparticles and nanocapsules. For some of those usages degradability of material is a disadvantage [87,88,89,97], for others, it has to be possible to strictly control the moment and way of degradation [91,106]. Moreover, degradation products are of high importance due to their possible toxicity on cells surrounding applied biomaterial [98]. As a result, researchers are testing and projecting polyurethane materials to reduce or prevent their degradability by human and animal enzymes and to prevent the formation of toxic degradation products [100]. However, an outcome that showed possible degradability of polyurethanes, while unfavorable for the medical field, was a promising result for the environment [92,93].

Results of the medical approach led to multiple experiments on PU’s degradation being conducted with enzymes like lipases, cholesterol esterase or chymotrypsin [81,82,101]. In 2007, Gautam with coworkers proved that commercially available *Candida rugosa* lipase successfully degraded polyester polyurethane particles. Enzyme binding affinity on the polymer surface proved to be of high importance. An increase in diethylene glycol concentration could be observed after incubation. Those results show that the enzymatic degradation of polyurethanes can lead to the generation of valuable products [61].

Since a large part of PU production is based on polyether polyols, it is imperative to search for enzymes that target urethane bond instead of the ester bond. Magnin et al. tested a blend of esterase and amidase due to some similarity of the urethane bond to the amide bond. According to their results, it seems that amidase was able to hydrolyze the urethane bond to some extent. It was possible as a second step after esterase degradation rather than as an independent process [83]. Amongst enzymes not classified as esterases, there are Impranil degrading cutinases [84], and polyacrylic PU coating degrading pancreatin [102].

Although reactions in an aqueous environment are most preferred, some investigations check the possibility of conducting enzymatic PU degradation in organic solvents. Lipase from *Candida antarctica* was able to catalyze the hydrolysis of polyurethane containing an aliphatic polyester chain in toluene at 60 °C [86]. However, even if the water is used as the solvent, it demands lots of adjustments, as it seems that degradability highly depends not only on the tested enzyme but also on the reaction environment parameters [96].

#### 3.5.4. Polyurethane Modification

As most of the researches point to limited availability of urethane bond for enzymes, it might seem that only polymers with a degradable flexible chain could be disposed of in this way. However, the growing environmental problem and need for energy-consuming and high technology processing systems for the synthesis of polyurethanes containing petroleum-based polyols resulted in demand for materials based on renewable and biodegradable resources. Various researches indicate the high potential of polyols and isocyanates derived from vegetable oils such as soybean oil [107], rapeseed oil [4], castor oil [108,109], jatropha oil, cardanol oil, and palm oil [110]. Another source for petroleum-based polyols replacement can be bio-polyols derived from the liquefaction of lignocellulosic biomass in polyhydric alcohols [111]. Moreover, the addition of specific chain extenders and biodegradable aliphatic polyesters like poly (lactic acid) [112], poly (caprolactone) and poly (butylene succinate) leads to higher biodegradability of polyurethanes [113].

The main obstacle in the application of this path is the influence of the introduced molecule on final product properties. Carriço and her team tested the impact of some of the biobased polyols. Their studies showed that the addition of lignin to polyurethane foam leads to an increase in the density and the decrease in thermal stability with the growing lignin content. Furthermore, higher amounts of castor oil led to the rise in density as well as the compressive strength of foam [114]. Preparation of polyurethane elastic foams containing various rapeseed oil-based polyols led to higher foams hysteresis, support factor and hardness [115]. Bio-polyols used in PU synthesis can be further modified to obtain the required properties of the final product. Modification of castor oil with maleic anhydride led to the different material structure—it was more closed and collapsed [109].

Biodegradable additives or substitutes in polyurethane synthesis make those polymers much more environmentally friendly due to both reduction of recalcitrant waste and possible application of milder reaction conditions [110]. However, for every novel biodegradable material, lots of testing is needed due to the impact of substitutions and additions on a broad spectrum of properties. In addition, positive changes in some of the characteristics can go hand in hand with detrimental changes in others.

## 4. Conclusions

There are lots of ways to deal with polyurethane waste, but they all still have a lot of room for improvement (Table 5). Landfilling is currently the most applied way to dispose of polyurethane waste, nonetheless, it is environmentally unfriendly, land consuming, and to a significant extent is economically unjustified. Another often used method is mechanical recycling. It is relatively cheap, but it has a lot of limitations. It also gives products that have much lower prices than the original polyurethane material. Chemical and feedstock recycling requires high temperatures and aggressive reagents, and at the moment, only one of those processes is applied on a larger scale. Biological degradation demands a moderate temperature, and does not require any dangerous chemicals, but it is still a long way until it could be applied on a technological scale Notably, biodegradation is most promising due to a wide range of possibilities and available modifications. Furthermore, using properly modified building blocks during PU synthesis results in degradable polymers, and combining those two approaches can be considered as a feasible solution for polyurethane waste accumulation.

## Figures and Tables

**Table 1:** Advantages of polyurethanes against chosen materials.

Rubber	Metal	Plastic
High abrasion resistance	Lightweight	High impact resistance
High cut and tear resistance	Noise reduction	Elastic memory
Superior load bearing	Abrasion resistance	Abrasion resistance
Thick section molding	Less expensive fabrication	Noise reduction
Colorability	Corrosion resistance	Variable coefficient of friction
Oil resistance	Resilience	Resilience
Ozone resistance	Impact resistance	Thick section molding
Radiation resistance	Flexibility	Lower cost tooling
Broader hardness range	Easily moldable	Low temperature resistance
Castable nature	Non-conductive	Cold flow resistance
Low pressure tooling	Non-sparking	Radiation resistance

**Table 2:** Comparison of chemical polyurethane recycling methods.

Treatment	Input	Output	Large Scale Application
Hydrolysis	EOL products production scraps	polyols, amine intermediates	No
Hydroglycolysis	EOL products, production scraps	polyols	No
Aminolysis	only foams	bi- or polyfunctional amines and alcohols	No
Phosphorolysis	production scraps	phosphorus containing oligouretanes	No
Glycolysis	only foams, segregated for rigid and flexible	polyols	Yes
Gasification	EOL products, production scraps	syngas	Yes
Pyrolysis	EOL products, production scraps	oil, gas, ash	No
Hydrogenation	EOL products, production scraps	gas, oil	No

EOL–End of life.

**Table 3:** Bacterial and fungal strains degrading different kinds of polyurethane.

	Microorganism Type	
	Bacteria	Fungi
Polyester PU coating (including Impranil^®^)	*Alicycliphilus* sp. [46] *Arthrobacter calcoaceticus* [47] *Acinetobacter garnei* [48] *Arthrobacter globiformis* [47] *Bacillus subtilis* [49,50] *Bacillus pumilus* [51] *Commamonas acidovorans* [52] *Pseudomonas aeruginosa* [47] *Pseudomonas cepacian* [47] *Pseudomonas chlororaphis* [53] *Pseudomonas fluorescens* [54] *Pseudomonas putida* [47,55]	*Aureobasidium pullulans* [56] *Cladosporium* sp. [56] *Cladosporium asperulatum* [57] *Curvularia senegalensis* [56] *Fusarium solani* [56] *Penicillium chrysogenum* [57] *Pestalotiopsis microspore* [58]
Polyester PU foam	*Alycycliphilus* sp. [59] *Pseudomonas aeruginosa* [60] *Pseudomonas chlororaphis* [61]	
Thermoplastic polyester PU	*Arthrobacter* sp. [62] *Bacillus* sp. [62] *Comamonas acidovorans* [63,64,65] *Corynebacterium* [60] *Micrococcus* sp. [62] *Pseudomonas* sp. [62] *Pseudomonas aeruginosa* [62,66,67,68]	*Alternaria* sp.*,* [69] *Alternaria solani* [70] *Aspergillus flavus* [71] *Aspergillus fumigatus* [72] *Aspergillus section flavi* [69] *Aspergillus tubigensis* [73] *Chaetomium globosum* [74] *Gliocladium roseum* [75] *Penicillium* sp. [69]
Thermoplastic polyether PU	*Staphylococcus epidermidis* [76]	
Polyether PU foam		*Alternaria* sp. [77] *Aspergillus fumigatus* [57] *Cladosporium tenuissinum* [57] *Cladosporium asperulatum* [57] *Cladosporium herbarum* [78] *Cladosporium montecillanum* [57] *Cladosporium pseudocladosporoides* [57] *Penicillium chrysogenum* [57]

**Table 4:** Enzymes degrading different kinds of polyurethane.

Type of PU	Enzyme
Polyester PU (Impranil)	Esterase [49,52,82,83] Lipase [53,54,81,82] Protease [82] cutinase [84]
Thermoplastic polyester PU	Lipases [85,86] Esterases [63,83,85] Pancreatin [87] Polyamidase [15] Proteases [87,88]
Thermoplastic polyether PU	Esterase [88,89] Chymotrypsin [88,89,90] Proteases [88,89,90,91]
Thermoplastic Polycarbonate PU	Cholesterol esterase [92,93]
Thermoplastic poly (ester ether) PU	Chymotrypsin [94]
Thermoplastic poly (ester urea) PU	Lipase [95] Cholesterol esterase [96,97,98]
Thermoplastic poly (ether urea) PU	Cholesterol esterase [97] Elastase [99] Papain [100]
Polyester PU coating	Lipase [101]
Polyacryl PU coating	Pancreatin [102]

**Table 5:** Comparison of polyurethane recycling and degradation methods.

	Large Scale Application	Reaction Conditions	Slabstock Recovery Possibility	Environmental Impact
Landfilling	Yes	Moderate	No	High land usage, possibility of fire, Accumulation in environment Can be applied for postconsumer waste
Mechanical recycling	Yes	Moderate to extreme	No	Applied only to postproduction waste Some of processes demand high temperature and pressure
Chemical recycling	Only glycolysis	Extreme	Yes	Demands high temperatures and pressure Organic solvents, catalysators, and other potentially dangerous chemicals are needed Can be applied for postconsumer waste
Energy recovery	Yes	Extreme	No	Toxic fumes release Ashes need to be landfilled Can be applied for postconsumer waste
Biological degradation	No	Moderate	Yes	Possibility of toxic compounds release Can be applied for postconsumer waste Can lead to complete mineralization of waste Can be applied on already existing landfills
